# Cincinnati Journal of Health

**Published:** 1845-03

**Authors:** 


### CINCINNATI JOURNAL OF HEALTH.

Our excuse for not sooner noticing this modest but meritorious little journal, is, simply, that we did not know of its existence. It is conducted by the "Physicians of the Cincinnati Dispensary and Vaccine Institution," and is published monthly, each number containing sixteen pages, 8vo., double columns. The object of the journal is to diffuse a knowledge of the laws that govern health, and of the causes that produce disease. It explains briefly various points of

Anatomy and Physiology; demonstrates such sources of disease as may be avoided by the community; discusses the subjects of dress, diet, and exercise; shows the effects and tendencies of improper modes of taking medicines; points out the antidotes for poisons, &c. &c.; all of which is couched in language suitable to the popular understanding. In a word, it is intended to make the journal a welcome and appropriate family vistor. It deserves to be extensively patronized, and we trust that it is. C.

